# Protein phosphorylation: A potential target in glioma development

**DOI:** 10.1002/ibra.12038

**Published:** 2022-05-08

**Authors:** Yu Pi, Chang‐Le Fang, Zhang‐Yu Su

**Affiliations:** ^1^ Department of Anesthesiology Southwest Medical University Luzhou Sichuan China

**Keywords:** glioblastoma (GBM), glioma, invasion, phosphorylation, proliferation

## Abstract

Glioma is one of the most common primary brain tumors, and mortality due to this disease is second only to cardiovascular and cerebrovascular diseases. In traditional surgery, it is difficult to eradicate glioma; often recurrence increases its malignant degree, leading to a large number of patients killed by this disease. It is one of the most important subjects to study its pathogenesis and explore effective treatment methods. Research on glioma mechanisms mainly focuses on the effect of DNA methylation in epigenetics. Although there are many studies on protein phosphorylation, there is no overall regulatory mechanism. Protein phosphorylation regulates a variety of cell functions, such as cell growth, division and differentiation, and apoptosis. As a consequence, protein phosphorylation plays a leading part in various activities of glioma, and can also be used as a target to regulate the development of glioma. This review is aimed at studying the effect of protein phosphorylation on glioma, understanding the pathological mechanism, and an in‐depth analysis of it. The following is a discussion on glioma growth, migration and invasion, resistance and death in phosphorylation, and the possibility of treating glioma by phosphorylation.

## Graphical abstract

Glioma is a common primary brain tumor, and its mortality rate is extremely high. Protein phosphorylation regulates a number of cellular functions like cell growth, differentiation, apoptosis, and cell signaling, so it can also be used as a target to regulate the development of glioma. This review is a discussion on the growth, migration and invasion, resistance, and death of glioma in phosphorylation and the possibility of treating glioma by phosphorylation.

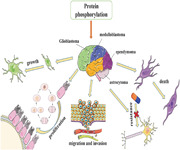

## 1 INTRODUCTION

### 1.1 Glioma

According to the histopathological and clinical characteristics, there are four grades of glioma (I, II, III, and IV). Grade I is benign glioma. The second grade is between the benign and malignant boundary of the glioma. Grade III above belongs to malignant glioma. Malignant gliomas grow fast and lead to more severe brain edema. There is no exactly effective treatment for glioma. In spite of temozolomide (TMZ) chemotherapy, which increases long‐term survival, treatment failure, and rapid glioma recurrence remain common.^1^ Moreover, despite undergoing the comprehensive treatment of surgery, radiotherapy, and chemotherapy, low‐level glioma is isolated from the normal brain region from mechanism and vascular mechanism. It makes the peripheral normal brain area denervation regulation and no longer be adjusted by the normal regulation. What is worse is that high‐grade glioma patients have a shorter survival period, with an average survival period of less than 1 year. Glioma is also divided into oligodendroglioma, astrocytoma, medulloblastoma, glioblastoma (GBM), ependymoma, and choroid plexus papilloma. GBM is the most common one, which is a Grade IV astrocytoma. Owing to its aggressiveness, it cannot be completely resected,^2^ and has a high mortality rate.^3^

### 1.2 Protein phosphorylation

**Figure 1:**
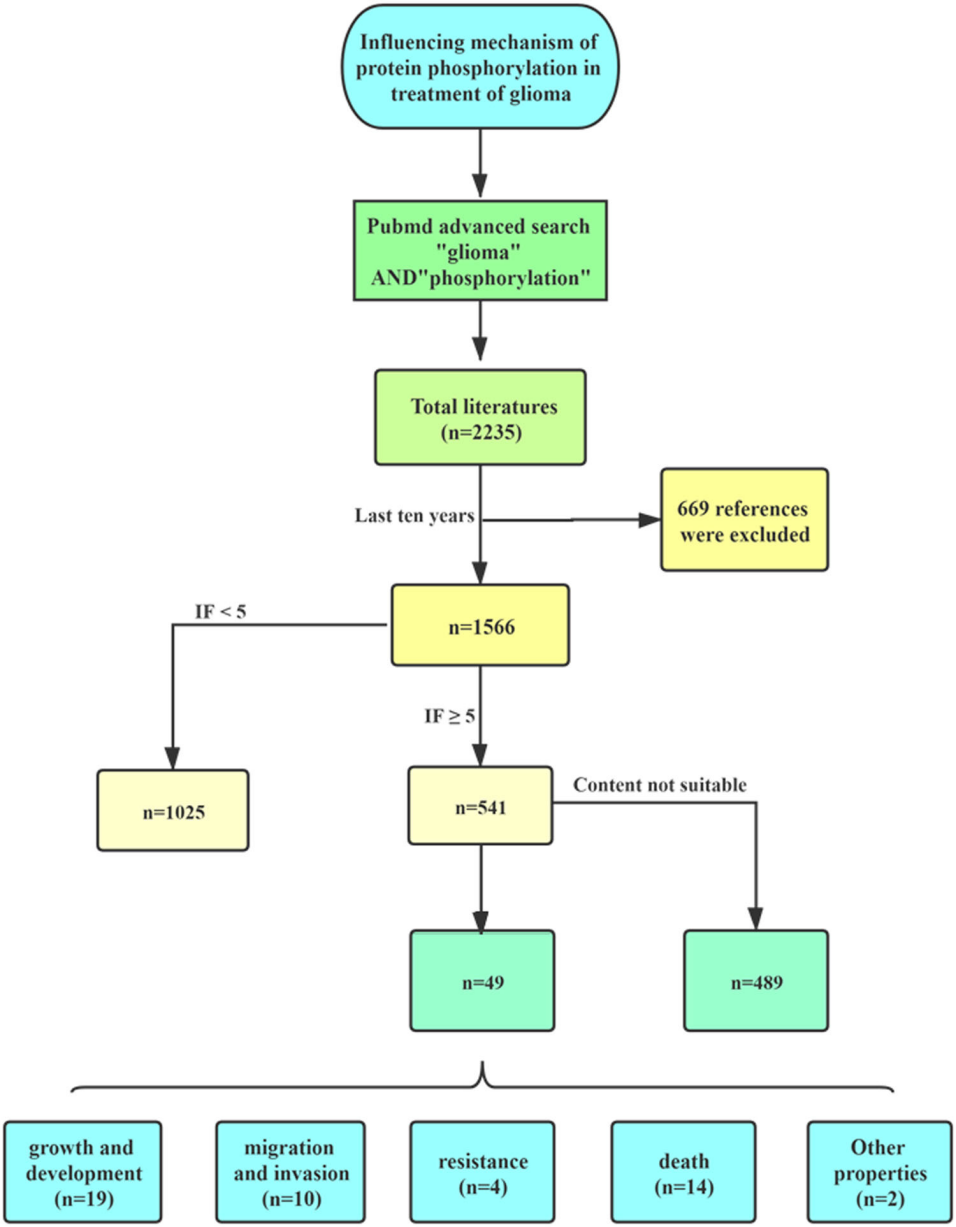
The literature screening flow chart of mechanisms by which phosphorylation affects glioma. *N* in the figure represents the number of literature. [Color figure can be viewed at wileyonlinelibrary.com]

Phosphoric acidification of protein is very common. Protein phosphorylation is necessary for the initiation of many biological phenomena, including cell growth, proliferation, apoptosis, and so on, because phosphorylation can directly regulate various aspects of protein function.^4^, ^5^ Regulated strictly by protein phosphatases and protein kinases, once the regulation of protein phosphorylation is disordered, it often leads to serious diseases, for example, cancer and glioma.^6^ Altering phosphorylation in any of the proteins is possibly connected with glioma, and they could be used as the potential targets for drug development for the treatment of glioma. Thus, drugs that target the phosphorylation pathway represent a hopeful area for glioma therapy. The literature on protein phosphorylation and glioma has been screened on PubMed (Figure 1).

## 2 THE REGULATORY ROLE OF PHOSPHORYLATION AS A TARGET IN GLIOMA DEVELOPMENT

### 2.1 Phosphorylation and the growth and development of glioma

#### 2.1.1 The occurrence of glioma

The occurrence of glioma is the cause. If we control the occurrence, the glioma will decrease. *KDM1A* stabilization is related to the occurrence of glioma. Glycogen synthase kinase‐3β (GSK3β) phosphorylates *KDM1A* serine 683 (Ser683) upon priming by CK1α, leading to the stabilization of *KDM1A*, which, in turn, leads to cancer stem cell self‐renewal and GBM tumorigenesis.^7^ Polarized M2 macrophages enhance the phosphorylation of *PGK1* threonine 243 (Thr243) by secreting interleukin‐6. In addition, inhibition of *PGK1* T243 phosphorylation abolishes macrophage‐promoted tumorigenesis and glycolysis.^8^ Therefore, phosphorylation of *PGK1* T243 may also be connected with the prognosis and malignancy of GBM. It has been confirmed that Atoh1 reduced the incidence of glioma and extended survival. Tyrosine 78 undergoes phosphorylation probably due to a Jak2‐mediated pathway. Phosphorylation of tyrosine 78 makes Atoh1 more stable and enhances the transcriptional activity of Atoh1.^9^ To sum up the above, restraining phosphorylation of Jak2‐mediated tyrosine 78 could reduce significantly glioma occurrence. EGFR/SRC/ERK signaling stabilizes *YTHDF2* protein by phosphorylating YTHDF2 Ser39 and Thr381 to promote tumorigenesis of GBM cells.^10^ The phosphorylation of *KDM1A* Ser683, *PGK1* T243, Atoh1 tyrosine 78, *YTHDF2* Ser39, and Thr381 can promote tumorigenesis, and if we can effectively control the phosphorylation of these proteins, we can prevent the development of glioma. Related literature is shown in Table 1.

**Table 1:** Literatures on protein phosphorylation and the occurrence of glioma

Reference	Impact factor	Fluency indices	Intervening measure	Result
Zhou et al.^7^	17.7280	Phosphorylation of KDM1A	GSK3 inhibitor (tideglusib)	Sensitizes mouse xenografts to chemotherapy and improved survival through KDM1A downregulation. Phosphorylation and deubiquitination of KDM1A in combination with USP22 led to gliomagenesis and tumor stem cell proliferation.
Zhang et al.^8^	14.5480	PGK1 T243 phosphorylation	Nothing	Inhibition of PGK1 T243 phosphorylation can promote tumorigenesis and disrupt the therapeutic potential of the connection between macrophages and tumor cells.
Klisch et al.^9^	7.5510	Atoh1's transcriptional activity	Nothing	Tyrosine 78 phosphorylation increased the transcriptional activity of Atoh1. Inhibiting Jak2‐mediated tyrosine 78 phosphorylation could affect tumor occurrence.
Fang et al.^10^	11.8780	LXRα and HIVEP2	Nothing	EGFR/SRC/ERK signaling stabilizes YTHDF2 protein by phosphorylating YTHDF2 serine 39 and threonine 381 to promote tumorigenesis of GBM cells.

Abbreviations: EGFR, epidermal growth factor receptor; ERK, extracellular signal‐regulated kinase; GBM, glioblastoma; GSK, glycogen synthase kinase.

#### 2.1.2 The growth of the glioma

**Figure 2:**
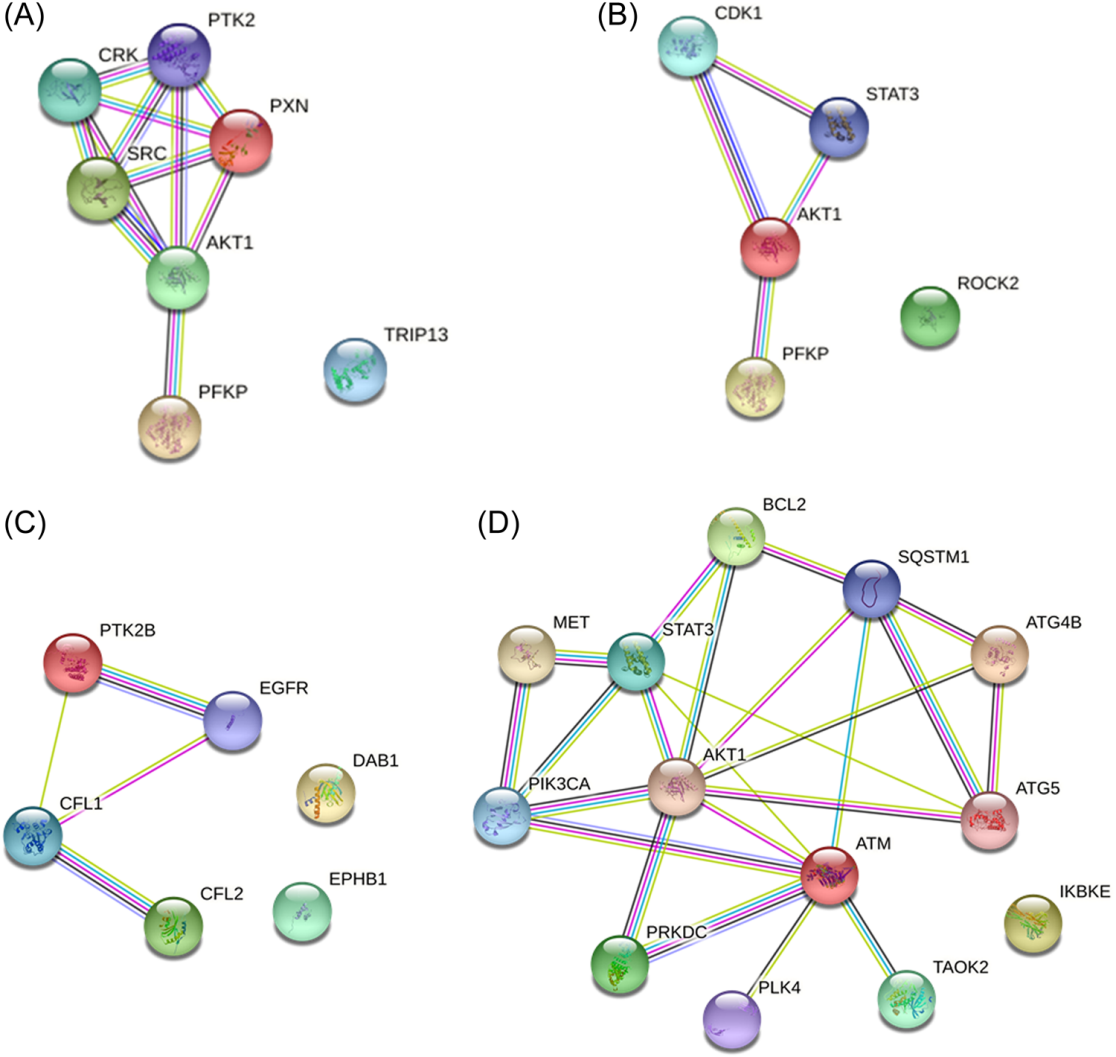
The protein interaction network. (A) The protein interaction network of related regulatory proteins in which phosphorylation affects glioma growth. (B) The protein interaction network of related regulatory proteins in which phosphorylation affects glioma proliferation. (C) The protein interaction network of related regulatory proteins in which phosphorylation affects glioma migration and invasion. (D) The protein interaction network of related regulatory proteins in which phosphorylation affects glioma apoptosis and autophagy. [Color figure can be viewed at wileyonlinelibrary.com]

After reading a lot of related literatures, I have listed a few briefly, from which we can find the relevance of phosphorylation and glioma growth. Phosphorylation has an influence on the size of glioma. A small‐molecule inhibitor (Lck‐I) was used to inhibit Lck phosphorylation, which blocked the phosphorylation of Paxillin and Crk‐II. Lck‐I could be used to treat human glioma stem cells, and it significantly inhibited self‐renewal, tumorsphere formation, and glioma size.^11^ Overexpression of *SAE1* induces increased SUMOylation and Ser473 phosphorylation of AKT, which stimulates the growth of glioma cells in vitro and in a nude mouse tumor model.^12^ Src phosphorylation has a positive role in the growth rate of glioma cells. Moreover, several tumor cell growth inhibitors decrease autophosphorylation of FAK and delay the GBM cell cycle progression.^13^ The phosphorylation of PFK1 platelet isoform (*PFKP*) S386 not only increased its expression but also promoted aerobic glycolysis and brain tumor growth.^14^ Elimination of *TRIP13* Y56 phosphorylation significantly reduced epidermal growth factor receptor (EGFR) signaling and GBM cell growth.^15^ The network of the above growth‐related regulatory proteins was analyzed by the STRING online platform (Figure 2A). The results show that, except *TRIP13*, all the above proteins were directly or indirectly related, especially ATK1, which was closely related to other proteins. Moreover, the above regulatory protein phosphorylation has positive significance for the size, morphology, and growth rate of glioma, so we can achieve the effect of limiting glioma growth by inhibiting the phosphorylation of one or more of the above proteins. Related literature is shown in Table 2.

**Table 2:** Literatures shown the connection between protein phosphorylation and the growth of glioma

Reference	Impact factor	Fluency indices	Intervening measure	Result
Zepecki et al.^11^	6.6340	Tumor size	Lck‐I	Lck‐I blocks the phosphorylation of Lck, which, in turn, inhibits the formation of pseudopods in glioma cells, thereby controlling not only migration but also the formation of tumorspheres.
Yang et al.^12^	5.1110	SAE1 expression level, SUMOylation, and Ser473	Regulate SAE1	Both SAE1 overexpression and silencing induced a corresponding increase or decrease in the phosphorylation of SUMOylation and Ser473, thereby promoting or inhibiting glioma cell growth.
Li et al.^13^	6.0540	Cell viability	Several tumor cell growth inhibitors	Reduces autophosphorylation of FAK, halts cells in the G2/M phase of the cell cycle, and delays the progression of cell growth and proliferation.
Hu et al.^15^	6.5080	GBM cell growth	Abrogating TRIP13 Y56 phosphorylation	Dramatically attenuate EGFR signaling and GBM cell growth.
Lee et al.^14^	11.8780	PFKP expression	Nothing	PFKP S386 phosphorylation increased PFKP expression, promoting glycolysis, tumor growth, and proliferation, and poor patient prognosis.

Abbreviations: EGFR, epidermal growth factor receptor; GBM, glioblastoma.

#### 2.1.3 The proliferation of glioma

All cells can proliferate, hence we need to prevent the proliferation of glioma cells to slow the progression of the tumor. *PFKP* S386 phosphorylation could increase cell proliferation.^14^ Eukaryotic cells generally proliferate by mitosis. Regulation of Olig2 mitosis and glioma function requires phosphorylation of three serine motifs (S10, S13, and S14) at the amino terminus.^16^ *PKMYT1* and *WEE1* restrain cell cycle protein B‐CDK1 activity through the phosphorylation of CDK1‐Y15 and accelerate timely completion of mitosis in GBM stem‐like cells (GSCs).^17^ Reduced cyclin D1 levels, selective downregulation of Akt Ser473 phosphorylation, and VEGF receptor levels in parkin‐expressing glioma cells promote G (1) phase cell cycle arrest and slow down the proliferation rate of glioma cells.^18^ Similarly, MiR‐29a/b/c induces G1 blockade by blocking AKT phosphorylation to the extent that it inhibits tumor cell proliferation.^19^ Therefore, phosphorylation is closely associated with mitosis in glioma cells. Fasudil, a ROCK2 phosphorylation inhibitor, significantly inhibited the proliferation of TMZ‐resistant gliomas.^20^ STAT3 tyrosine phosphorylation has been proved to accelerate the growth and glioma cell proliferation ensuing in significant long‐term survival. Downregulating or inhibiting phosphorylation of Akt1 and NF‐κB‐p65 could lower glioma cell proliferation.^21^, ^22^, ^23^ Knockout of TRIM22 decrease IKKα/β and IκBα phosphorylation and reduce GBM cell proliferation.^24^ The phosphorylation of PFKP, serine motif (S10, S13, and S14), CDK1, Akt, ROCK2, STAT3, IKKα/β, and IκBα could improve glioma proliferation. According to the protein interaction network, we know that all the proteins except ROCK2 have a strong interactive relationship (Figure 2B). To sum up, we can inhibit glioma mitosis and proliferation rate by adapting protein phosphorylation. Related literature is shown in Table 3.

**Table 3:** Literature shows that protein phosphorylation affects the proliferation of glioma

Reference	Impact factor	Fluency indices	Intervening measure	Result
Lee et al.^14^	11.8780	PFKP expression	PFKP S386 phosphorylation	Increase PFKP expression and promotes cell proliferation.
Zhou et al.^16^	7.8150	Phosphorylation of S10, S13, and S14	Nothing	Phosphorylation of amino‐terminal S10, S13, and S14 regulates the mitosis of Olig2 and also controls the growth of glioma cells.
Toledo et al.^17^	7.8150	Cyclin B‐CDK1 activity	Genome‐wide CRISPR‐Cas9 knockout	Inhibition of cell cycle protein B‐CDK1 activity through the phosphorylation of CDK1‐Y15 promotes mitosis in NSCs and may be glioma lethal in GSCs.
Yeo et al.^18^	8.3780	VEGF receptor, cyclin D1, and Akt phosphorylation	Establish a control group of wild‐type cells	Parkin expression decreased cyclin D1 and VEGF receptor levels in glioma cells, and Akt serine 473 phosphorylation was selectively downregulated, promoting G(1) phase block and inhibiting proliferation.
Shi et al.^19^	5.9590	AKT and GSK‐3β phosphorylation	Nothing	The potential therapeutic mechanism of miR‐29a/b/c in glioma is to inhibit tumor cell proliferation by downregulating cyclin D1 expression, phosphorylation of AKT, GSK‐3β, and thus inducing G1 blockade.
Zhang et al.^20^	5.9590	ROCK2 phosphorylation	ROCK2/moesin	Fasudil reduces glioma TMZ resistance by inhibiting ROCK2 phosphorylation, thereby inhibiting proliferation.
Wang et al.^21^	6.5080	Akt1 and NF‐κB‐p65 phosphorylation	IL‐17	IL‐17 induces increased phosphorylation of Akt1 and NF‐κB‐p65, which, in turn, promotes glioma proliferation.
Wang et al.^22^	5.7100	Expression of Akt, phosphorylated (p)‐Akt, NF‐κB	Knocking down miR‐19a/b	Downregulating phosphorylated p‐Akt inhibited cell proliferation.
Hou et al.^23^	8.0630	AKT phosphorylation and GSC proliferation	AEBP1 knockdown or ACT001 or PI3K inhibitor	Knockdown of ACT001 target AEBP1 inhibits AKT phosphorylation and tumor cell proliferation to exert antitumor effects.
Ji et al.^24^	8.0860	IKKα/β, IκBα, and TRIM22	Knockout of TRIM22	TRIM22 knockdown decreased IKKα/β and IκBα phosphorylation, reduced GBM cell proliferation, as well as removed the progrowth effect.

Abbreviations: GSC, GBM stem‐like cell; GSK, glycogen synthase kinase; NF‐κB, nuclear factor‐κB; TMZ, temozolomide.

### 2.2 Phosphorylation and glioma migration and invasion

Inside the brain, glioma cells migrate rapidly according to the extracellular environment and invade surrounding structures. The reason why *N*‐acetylgalactosaminyltransferase 2 (*GALNT2*) was closely associated with the migration and invasion of glioma cells is that *GALNT2* facilitated the malignant features of gliomas by affecting the O‐glycosylation, EGFR phosphorylation, and subsequently the downstream PI3K/Akt/mTOR axis.^25^ Thus, *GALNT2* knockout decreases the level of phosphorylated EGFR, which could improve the malignant characteristics of glioma. Hence *GALNT2* may serve as a potential target of action to treat glioma in the future. Small interfering RNA‐mediated *WNK1* or *OSR1* knockdown reduced glioma migration by eliminating NKCC1‐regulated phosphorylation activation.^26^ Similarly, RELN regulated the migration of GBM cells through *DAB1* tyrosine phosphorylation.^27^ The major feature of GBM is the invasion of the healthy brain parenchyma. The invasion of glioma is always what we value most. Heparin‐binding EGF (HB‐EGF) promoted EGFRvA Y845 site and STAT3 phosphorylation, which generated a positive feedback loop and it may enhance invasive function.^28^ Actomyosin is closely related to the invasive function of glioma. Myosin light chain 2 expression and phosphorylation are closely connected to MerTK activity, suggesting that the role of MerTK in glioma cell invasion is mediated through the contractility of actin. The receptor MerTK overexpressed and increased invasive potential in GBM.^29^ KAP low expression activates Cdk2, thereby promoting invasion through increased retinoblastoma phosphorylation and inactivation of the actin inhibitors mediated by Cdc2.^30^ Chronophin protein expression has an inverse association with phosphorylation level of cofilin in glioma cells. Chronophin‐deficient GBM cells revealed elevated cofilin phosphorylation, increased polymeric actin, and higher cell migration directionality, and they improved in vitro invasiveness.^31^ Besides, phosphorylation of proline‐rich tyrosine kinase 2 (Pyk2) is involved in the mechanism of store‐operated Ca (2+) entry (SOCE) regulating adhesive foci transformation and epithelial–mesenchymal (like) transformation in glioma cells.^32^ What can be seen from this is that the SOCE‐Pyk2 pathway is essential for migration and invasion of glioma. Ephrin‐B2/Fc‐induced phosphorylation of EphB1 significantly reduced migration and invasion.^33^ In addition, a class of glioma cells has unphosphorylated OLIG2 (S10, S13, and S14), yet these cells are highly migratory and invasive in vitro and in vivo. The mechanism is that unphosphorylated *OLIG2* induced *TGF*‐β2 expression and promoted the aggressive mesenchymal properties of glioma cells.^34^ Protein–protein interaction network of the above‐related regulatory proteins was built (Figure 2C). When phosphorylated, each protein can promote or restrain glioma migration and invasion. Therefore, we can regulate glioma migration and invasion through protein interactions. Related literature is shown in Table 4.

**Table 4:** Protein phosphorylation affects the migration and invasion of glioma

Reference	Impact factor	Fluency indices	Intervening measure	Result
Sun et al.^25^	5.2370	O‐glycosylation and phosphorylation of EGFR	GALNT2 knockdown and overexpression	GALNT2G knockdown or overexpression promotes or inhibits O‐glycosylation and phosphorylation of the epidermal growth factor receptor, respectively, to affect the malignant properties of gliomas.
Zhu et al.^26^	10.6790	NKCC1, WNK1, OSR1, K^+^, and Cl^−^ content	Knockdown of WNK1 or OSR1	Reduce K^+^ and Cl^−^ concentrations intracellularly, increase NKCC1, WNK1, and OSR1 phosphorylation, and enhance glioma migration.
Schulze et al.^27^	6.1550	Migration	RELN silence	Regulate glioblastoma cell migration.
Zhou et al.^28^	8.3780	Expression of STAT3, HB‐EGF, and EGFRvA	Nothing	EGFRvA induced STAT3 expression, which, in turn, upregulated HB‐EGF expression, and HB‐EGF feedback prompted EGFRvA Y845 site and STAT3 site phosphorylation, which may enhance invasive function.
Wang et al.^29^	6.6340	Invasive capacity, MerTK expression	Concomitant downregulation of Nestin and Sox	MerTK expression diminishes significantly. MerTK was associated with myosin light chain 2 expression and phosphorylation, which affects invasive function.
Li et al.^30^ (2015)	6.6340	Retinoblastoma phosphorylation, E2F‐dependent Cdc2 expression	Decrease KAP expression	Activation of Cdk2 increases retinoblastoma Cdc2 expression and phosphorylation, which induces caldesmon inactivation to promote invasion.
Schulze et al.^31^	6.6340	Cofilin phosphorylation, polymerized actin	Decrease chronophin protein expression	Chronophin deficiency elevated cofilin phosphorylation, directional cell migration, and invasiveness to the point of reducing patient cure rates.
Zhu et al.^32^	5.6460	Orai1, Ca (2+) concentration	Inhibition SOCE or Orai1 downregulation	Phosphorylation of Pyk2 is involved in the mechanism by which SOCE affects the migration and invasion of glioma cells.
Teng et al.^33^	10.0910	EphB1 and EphB1 phosphorylation	Overexpress EphB1	EphB1 overexpression did not affect external cell migration and invasion. However, EphB1 phosphorylation decreased migration and invasion.
Singh et al.^34^	7.8150	OLIG2, TGF‐β2 expression	Nothing	OLIG2, when unphosphorylated, drives TGF‐β2 expression and promotes glioma cell migration and aggressiveness.

Abbreviations: GALNT2, *N*‐acetylgalactosaminyltransferase 2; HB‐EGF, heparin‐binding EGF; Pyk2, proline‐rich tyrosine kinase 2; SOCE, store‐operated Ca (2+) entry.

### 2.3 Phosphorylation and the resistance of glioma

Glioma resistance could make chemotherapy fail. Current treatments also hope to bypass the resistance of tumor cells, thereby achieving a breakthrough. ACT001, originally an ancient anti‐inflammatory drug, was found to demonstrate anti‐GBM activity in clinical trials. ACT001 treatment can modulate antitumor immunity in GBM by inhibiting phosphorylated STAT3.^35^ Thus, STAT3 has a significant impact on glioma immunosuppression. The mechanism involved is that the tyrosine kinase receptor Tie2 physically interacted with FGFR1 to increase drug resistance expression in GSCs by promoting STAT3 phosphorylation and binding to the AURKA promoter.^36^ We can influence the immunity of glioma via phosphorylation. A combined ERK/mTOR targeted elimination inhibits GSK‐3β phosphorylation, leading to MAP1B phosphorylation and confers cellular sensitization.^37^ The above data represented molecular signaling networks for chronic inhibition of anti‐mTOR in cultured primary human GBM cells and point to novel therapeutic strategies. Plus, we can also reduce sensitivity. A novel N‐linked glycosylation inhibitor (NGI‐1) inhibited multiple receptor tyrosine kinase glycosylation and phosphorylation in GBM. NGI‐1 sensitized GBM in multiple models to chemotherapy and radiotherapy for therapeutic purposes and may be particularly effective in GBMs with preserved PTEN activity.^38^ Targeted therapy against PI3K has become a very popular treatment for GBM; nevertheless, the emergence of resistance limited treatment potential. Thus, to achieve a good therapeutic effect, the resistance of glioma can be affected by regulating protein phosphorylation. Related literature is shown in Table 5.

**Table 5:** Protein phosphorylation has an influence on the resistance of glioma

Reference	Impact factor	Fluency indices	Intervening measure	Result
Tong et al.^35^	8.0630	STAT3	Western blot analysis, RT‐PCR, and immunofluorescence	Inhibiting phosphorylated STAT3 can modulate antitumor immunity in GBM.
Li et al.^36^	8.3780	Aurora A expression	Tie2 and FGFR1	Promotes STAT3 phosphorylation and binds AURKA, leading to Aurora A expression in drug‐resistant tumor cells, thereby promoting the expression of drug resistance.
Laks et al.^37^	10.0910	ERK/mTOR, MAP1B	Primary, human GBM cell cultures	Abrogates inhibitory GSK3B phosphorylation, leading to MAP1B phosphorylation and sensitization, and develops resistance to mTOR inhibitors.
Wahl et al.^38^	8.9110	Phosphorylation of multiple receptor tyrosine kinases	NGI‐1	NGI‐1 inhibits tyrosine kinases glycosylation and phosphorylation, making chemotherapy and radiotherapy more sensitive.

Abbreviations: GSK, glycogen synthase kinase; RT‐PCR, reverse transcription‐polymerase chain reaction.

### 2.4 Phosphorylation and the death of glioma cell

The death of glioma cells includes apoptosis and necrosis. Glioma cell death often interferes with the experiment. *PLK4* interacted with IKBKE and thus phosphorylated it, resulting in an increase in NF‐κB antiapoptosis in GBM cells.^39^ *SAE1* silence obviously suppressed the phosphorylation of Akt SUMOylation and Ser473, induced a G2 phase block in the cell cycle, and led to apoptosis driven by a series of biochemical molecular events.^12^ ANXA2 increased the expression of miR155HG through STAT3 phosphorylation. Similarly, knocking down miR155HG induced the G1/S phase block, which, in turn, inhibited division and increased apoptosis.^40^ GBM 8401 cells transfected with wild‐type (WT) IKKα restrained zerumbone‐induced apoptosis as well as significantly reduced the levels of IKKα phosphorylation in a time‐dependent manner. Similarly, transfection of GBM 8401 cells with Akt showed a similar effect.^41^ Therefore, the conclusion is that followed by Akt and IKKα phosphorylation was involved in zerumbone‐induced apoptosis in GBM cells. Downregulation of cathepsin L resulted in a significant decrease in CD133 expression and a decrease in phosphorylation of ATM and DNA‐PKcs. Cathepsin L inhibition significantly reduced GSC growth and promoted apoptosis.^42^ Thus, it can be seen the phosphorylation of ATM and DNA‐PKcs is associated with cell apoptosis. Autophagy is a pretty typical form of death. Inhibition of AKT‐MTOR‐RPS6KB1 pathway induced GBM cells autophagy according to phosphorylation status.^43^ MST4 phosphorylated serine 383 of ATG4B, activated ATG4B, and increased cellular autophagic flux.^44^ Besides, if MDA‐9 is inhibited, GSCs will undergo cellular autophagic and die. MDA‐9 suppresses high levels of autophagy by phosphorylating BCL2 and EGFR signaling to achieve self‐protection.^45^, ^46^ Besides, regarding autophagy, PAK1‐mediated phosphorylation of ATG5 and phosphorylation of c‐MET and PI3K in GPCs under hypoxia conditions could affect cell autophagy.^47^, ^48^ There was an experiment that revealed that the phosphorylation level of p62/SQSTM1 was reduced in the presence of PIM knockdown. However, phosphorylated p62/SQSTM1 (S332E) abolished the effect of PIM inhibitors and the upregulation of apoptosis on the apoptosis‐inducing ligand (TRAIL)‐r2/DR5.^49^ Globally, it represents that p62/SQSTM1 (Ser332) phosphorylation regulates the TRAIL‐activated external apoptotic pathway and thus controls apoptosis. PKM2 also regulates apoptosis. PKM2 in mitochondria phosphorylates the Bcl2 T69 site, and the level of phosphorylation and conformational changes correlate with the grading and prognosis of malignant gliomas.^50^ It revealed a mechanism of effect that can directly inhibit apoptosis through phosphorylation of Bcl2. Moreover, circ‐MAPK4 regulates apoptosis in glioma cells by regulating p38/MAPK phosphorylation level.^51^ Above experiments confirmed that phosphorylation can adjust the death of glioma cells to improve the success rate of the experiment. The network of the above death‐related regulatory proteins was analyzed by STRING online platform (Figure 2D). The results showed that all the above proteins were directly or indirectly related. Thus, we can achieve the effect of controlling glioma death by regulating the phosphorylation of one or more of the above proteins. Related literature is shown in Table 6.

**Table 6:** Protein phosphorylation could regulate the death of glioma

Reference	Impact factor	Fluency indices	Intervening measure	Result
Zhang et al.^39^	6.5080	NF‐κB transcriptional activity	inhibition PLK4	Inhibition of PLK4 inhibits cell growth, and PLK4 phosphorylation with IKBKE promotes NF‐κB transcription and inhibits apoptosis.
Yang et al.^12^	5.1110	SUMOylated proteins, Ser473 phosphorylation	SAE1 overexpression or silence	SAE1 expression positively correlates with phosphorylation of Akt SUMOylation and Ser473 and controls the growth and malignancy of gliomas.
Wu et al.^40^	5.6460	miR155HG expression	Intracranial GBM mouse model; knock out miR155HG	ANXA2 promoted phosphorylation of STAT3 to upregulate miR155HG expression. Knocking down miR155HG led to blocking of the G1/S phase, which elevated the chance of apoptosis.
Weng et al.^41^	5.2030	Akt phosphorylation	Transfected IKK and Akt	Zerumbone significantly prevented phosphorylation of Akt. Akt and FOXO1 phosphorylation facilitated zerumbone‐mediated apoptosis in glioma cells.
Wang et al.^42^	6.5080	CD133 expression, phosphorylation of ATM and DNA‐PKcs	Set up control groups; knockdown of cathepsin L	Reduce CD133 expression and decrease phosphorylation of ATM and DNA‐PKcs. Histone L, combined with radiotherapy, can be targeted for inhibition and promotion of apoptosis.
Wang et al.^43^	11.0590	AKT‐MTOR‐RPS6KB1	Phosphorylation	Inhibited the AKT‐MTOR‐RPS6KB1 pathway and led to GBM cell autophagy.
Huang et al.^44^	23.9160	ATG4B activity, autophagic flux	Radiate	ATG4B phosphorylation stimulates ATG4B activity and increases autophagic flux.
Talukdar et al.^45^	11.0590	Phosphorylation of BCL2	SDCBP/MDA‐9/syntenin silence	Induce autophagic death in GSCs, promote phosphorylation of BCL2, and restrain autophagy protein‐ATG5, LAMP1, and LC3B overexpression.
Talukdar et al.^45^	9.5800	Phosphorylation of BCL2; pBCL2	Knock out MDA‐9	Decrease phosphorylation of BCL2, and pBCL2 is downregulated, and it induced GSCs' death.
Feng et al.^47^ (2021)	10.091	Levels of PAK1 (K420) acetylation, ATG5 (T101) phosphorylation	Hypoxia	The two were significantly correlated. Hypoxia induced ELP3‐mediated PAK1 acetylation and PAK1 induced phosphorylation of ATG5 to promote autophagy.
Cheng et al.^48^	8.063	Phosphorylation of c‐MET and PI3K	Hypoxic drug	c‐MET and PI3K phosphorylation in GPCs under hypoxic conditions was reduced by c‐MET inhibitor, leading to oxidative mutagenesis and apoptosis.
Serrano‐Saenz et al.^49^	5.9590	Phosphorylation of p62/SQSTM1	Knock out PIM	A decreased phosphorylation of p62/SQSTM1 and increased apoptosis.
Liang et al.^50^	17.8480	Phosphorylation of Bcl2	Nothing	Bcl2 phosphorylation inhibits apoptosis directly.
He et al.^51^	10.6790	Phosphorylation levels of p38/MAPK	Inhibiting miR‐125a‐3p	The upregulation of p38/MAPK phosphorylation could be slowed down to the extent that circ‐MAPK4‐induced apoptosis in glioma cells did not increase excessively.

Abbreviations: GBM, glioblastoma; GSC, GBM stem‐like cell; NF‐κB, nuclear factor‐κB.

### 2.5 Phosphorylation and other properties of glioma

Now let us observe other effects of protein phosphorylation on glioma. Tumor heterogeneity is an important feature of malignancy, which could significantly affect the therapeutic effects and prognosis. Glioma cells exhibited heterogeneous patterns of TGF‐β pathway activation with altered phosphorylation of SMAD2, SMAD3, and SMAD1/5/8.^52^ Proteomics, closely related to protein phosphorylation, has been widely used in the field of heterogeneity research. In addition, the stemness of glioma is the basis of its survival and proliferation and is the key to play the role of malignant biology. Studies have shown that hypoxia can enhance the stemness of gliomas. Hypoxia downregulated ID2 phosphorylation on Thr27 through inactivation of *DYRK1A* and *DYRK1B*. In contrast, high DYRK1 expression increases ID2 Thr27 phosphorylation, leading to glioma stemness deficiency, which inhibits tumor growth and is more favorable to the prognosis of GBM patients.^53^ To sum up, protein phosphorylation has an influence on the heterogeneity and stemness of glioma. Other properties remain to be further studied. Related literature is shown in Table 7.

**Table 7:** Literatures shown the effect of protein phosphorylation on other aspects of glioma

Reference	Impact factor	Fluency indices	Intervening measure	Result
Seystahl et al.^52^	10.0910	Phosphorylation of SMAD2, 3 and SMAD1/5/8	TGF‐β pathway	SMAD2,3 and SMAD1/5/8 phosphorylation showed heterogeneous patterns.
Lee et al.^53^	43.0700	Expression of DYRK1, phosphorylates of ID2	Hypoxia	Downregulates ID2 phosphorylation. Elevated expression of DYRK1 drives ID2 Thr27 phosphorylation, decreases HIF2α stability, and inhibits glioma cell growth and loss of tumor stem cells.

## 3 POSSIBILITY OF TREATMENT

**Figure 3:**
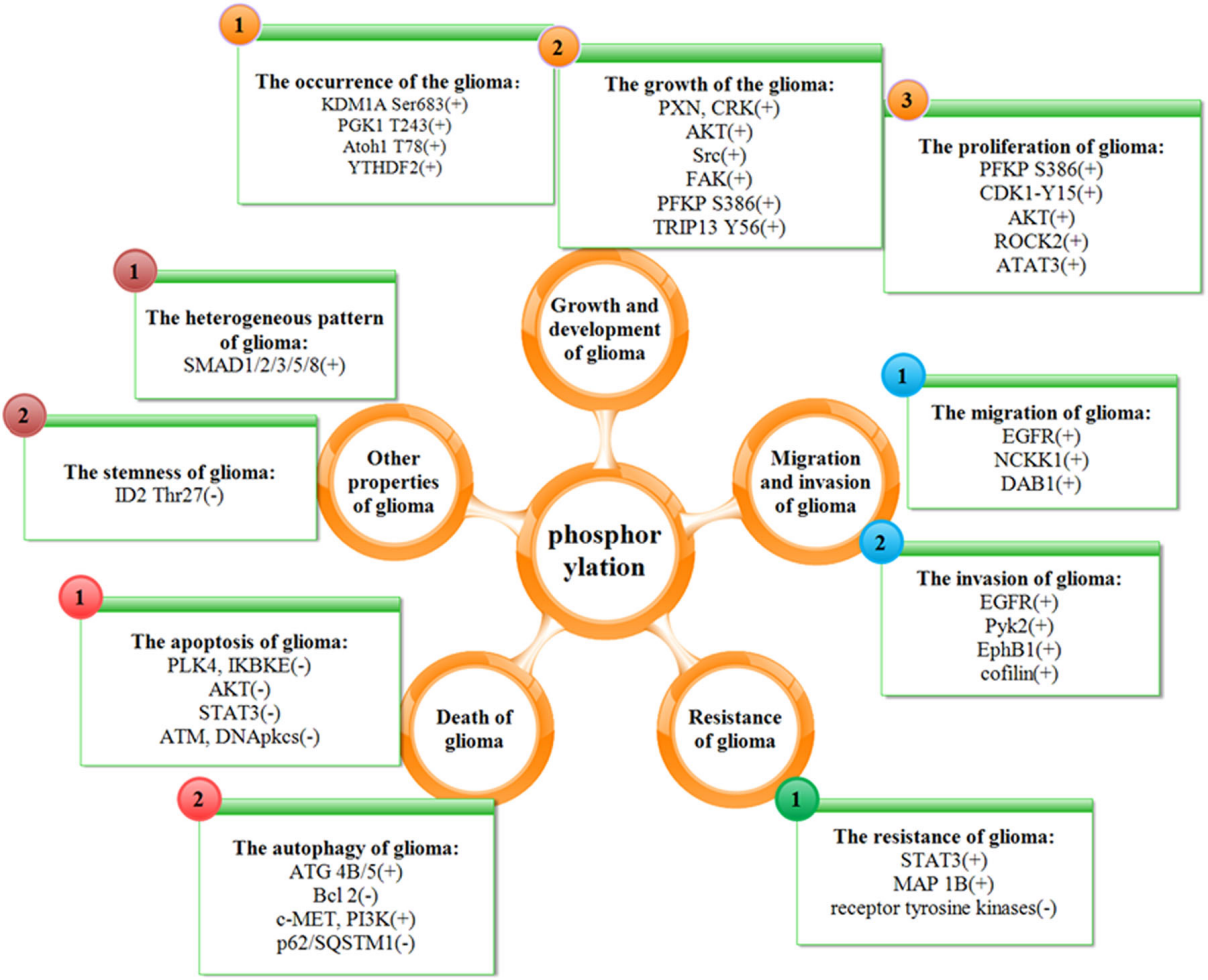
The structural diagram of the mechanism by which phosphorylation affects glioma (+) represents the promoting effect of protein phosphorylation on the properties of glioma. (−) represents the inhibitory effect of protein phosphorylation on the properties of glioma. [Color figure can be viewed at wileyonlinelibrary.com]

Currently, many drug studies refer to protein phosphorylation. Similarly, various treatments for glioma are associated with protein phosphorylation. We just need to figure out that phosphorylation has an influence on glioma, and find a way to break through from the aspect. We all know that GALNT2 is inextricably linked to the physiological processes of cell proliferation, migration, and invasion in gliomas. But the mechanism by which GALNT2 promotes the malignant features of gliomas is by affecting EGFR phosphorylation.^25^ Concurrent inhibition of p110β and JNK restrains the phosphorylation of Akt, FAK, and zyxin to inhibit the proliferation and migration of GBM cells.^54^ Besides, Histone phosphorylation plays an important regulatory role in DNA repair structure and transcription during cell proliferation and apoptosis.^55^ One of the major tasks of phosphoproteins is to provide potential biomarkers for either diagnosis or drug targets in medical applications.^56^ STAT3 phosphorylation can regulate the proliferation, migration, invasion, apoptosis, and other physiological processes of glioma, thus STAT is a good regulatory target, and the targeted inhibition of glioma by STAT phosphorylation has a good therapeutic prospect. The proteins AKT and FGFR, which appeared frequently in this paper, have become important therapeutic targets for glioma and improved the sensitivity of glioma by targeted inhibition of phosphorylation, thus playing an active role in the treatment of glioma.^57^ Phosphorylation of many proteins can affect the development of glioma (Figure 3). To conclude, protein phosphorylation will be a breakthrough, providing new ideas for the treatment of glioma. Therefore, it is entirely possible to treat glioma by phosphorylation.

## 4 CONCLUSIONS AND PROSPECTS

Protein phosphorylation is associated with the occurrence, growth, proliferation, migration, invasion, resistance, and death of glioma cells, while other properties of glioma cells remain to be further investigated. As is well known, phosphorylation and dephosphorylation of protein, which is a reversible process, almost regulate the cytoskeleton development and cell proliferation, differentiation, apoptosis, and all the process of life activity. Reversible protein phosphorylation is one of the main currently known ways of signal transduction. Therefore, we can effectively control glioma cells depending on protein phosphorylation. We just need to grasp the corresponding mechanism of influence to find a method to the problem. If we can make good use of protein phosphorylation, glioma will also have pretty treatment, not limited to surgery. Although at present, the targeted therapy of intracellular signaling pathways has not gained a great satisfactory effect, we believe that the protein phosphorylation targeted regulation can achieve great effect in the future; its targeted drugs can also be further developed and put into use in the clinic, which provides another way for the treatment of patients and has a bright prospect.

## AUTHOR CONTRIBUTIONS

Yu Pi wrote the manuscript. Chang‐Le Fang and Zhang‐Yu Su helped to modify the manuscript. All authors have read and approved the final manuscript.

## CONFLICT OF INTEREST

The authors declare no conflict of interest.

## ETHICS STATEMENT

Consent for publication was obtained from all authors.

## Data Availability

Data sharing is not applicable to this article as no new data were created or analyzed in this study.
